# Zinc Orthophosphate Can Reduce Nitrate-Induced Corrosion of Lead Solder

**DOI:** 10.1021/acsestwater.3c00786

**Published:** 2024-07-30

**Authors:** Kathryn G. Lopez, Jinghua Xiao, Christopher Crockett, Christian Lytle, Haley Grubbs, Marc Edwards

**Affiliations:** † Department of Civil & Environmental Engineering, Virginia Tech, Blacksburg, Virginia 24060, United States; ‡ Aqua America, Bryn Mawr, Pennsylvania 19010, United States

**Keywords:** contamination, drinking water, lead, particulate matter, phosphates

## Abstract

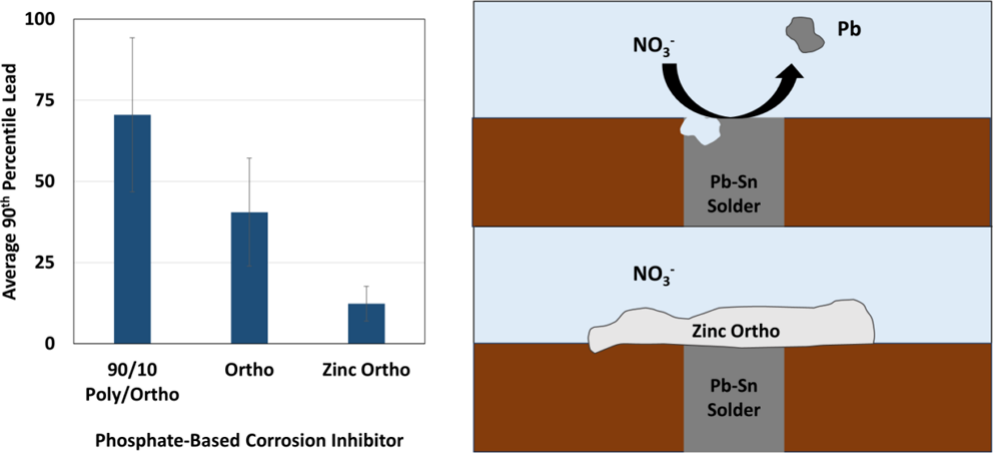

Nitrate-induced spallation of lead-bearing solder particles into drinking water is not sufficiently controlled by phosphate-based inhibitors, although adding zinc can improve their performance. Studies using copper coupons coated with new lead–tin solder in water with up to 12 mg/L nitrate demonstrated that zinc orthophosphate reduced lead release by more than 90% and outperformed orthophosphate alone. Lead release and spallation from harvested pipes with decades-old lead–tin solder in a high nitrate water were improved but not eliminated with zinc orthophosphate over a period of months. When applied at a water utility with high source water nitrate, monthly in-home field sampling showed that 90th percentile lead levels dropped below the action level after dosing zinc orthophosphate at full scale for 6 months. Scanning electron microscopy (SEM) analysis of pipe scales revealed that zinc and orthophosphate codeposit at the copper–solder interface and may act as a mixed inhibitor, with zinc inhibiting the cathodic reaction on the copper pipe, phosphate limiting the anodic reaction, and an added benefit of zinc orthophosphate preferentially precipitating at the galvanic interface between the anode and the cathode. Updates to corrosion control guidance for waters with higher nitrate due to seasonal runoff or source water changes are needed.

## Abstract 2

Zinc orthophosphate reduced nitrate-induced solder corrosion in laboratory studies and at a water utility. It was found to reduce anodic and cathodic corrosion while preferentially precipitating at the copper–solder interface.

## Introduction

A recent change in source from groundwater to surface water triggered high monthly 90th percentile lead levels of up to 230 μg/L in one midwestern community. Seasonal nitrate spikes in the surface water (up to 7.8 mg/L NO_3_–N) due to runoff following heavy rain events were found to initiate lead–tin solder corrosion in the distribution system, causing the unexpected high lead and the erratic spalling of large metallic solder particles from home plumbing.^1^ The elevated lead persisted even after application of a 90/10 ortho/poly phosphate blend and high doses of orthophosphate (up to 5.5 mg/L as PO_4_) for over 18 months. This discovery was worrisome because this mode of attack on lead solder was not recognized in a recent state of the art literature review on solder corrosion.^2^ Additionally, the U.S. Environmental Protection Agency (EPA) does not yet recognize nitrate as a noteworthy factor affecting corrosivity, and there is no prior research identifying appropriate corrosion control for this recently identified form of corrosion.^3,4^

Lead release from solder can be driven by galvanic corrosion between the large surface area of a cathodic copper pipe and a relatively small surface area of anodic solder. The nature of galvanic corrosion and the magnitude of the release are known to be affected by water chemistry parameters including alkalinity, the chloride-to-sulfate mass ratio (CSMR), pH, and corrosion inhibitors.^2,4−12^ Conventional phosphate corrosion inhibitors have often been used to reduce lead release from solder by passivating the anodic reaction, but there have been cases where phosphate actually worsened overall lead release for reasons that are not presently understood.^13^

Here, dosing of zinc in addition to orthophosphate is hypothesized to provide synergistic benefits due to mixed inhibition, in which zinc coats and inhibits the cathodic reaction to supplement passivation of the anode by orthophosphate. One prior study suggested that zinc orthophosphate best inhibited galvanic corrosion of lead solder in the presence of nitrate and that zinc acted as a cathodic inhibitor, but these findings were based on electrochemical data and no confirmatory measurements of reduced lead release to water or inhibitor deposition were obtained.^14^ While there are cases in which zinc orthophosphate demonstrated a superior ability to inhibit lead solder corrosion, the mixed inhibition mechanism has never been proven.^15,16^ Understanding how to control corrosion of lead–tin solder is crucial given that subtle changes in water chemistry, such as an increase in CSMR or nitrate in runoff water, can trigger major water lead contamination events.^17,18^

The recent documentation of an EPA Lead and Copper Rule (LCR) Action level exceedance at least partly due to high nitrate poses a serious concern because nitrate maximum contaminant level violations are common and the treatment options for its removal are costly. Global surface water nitrate levels have increased over the last 50 years, and despite efforts to improve water quality in the U.S., many areas of the country still have high source water nitrate levels.^19−21^ At the same time, more utilities are diversifying their source water portfolios to meet sustainability targets, which can include the use of recycled waters that will sometimes have higher nitrate.^22^ The inability to control elevated lead due to nitrate with 90 or 100% orthophosphate underscores the need for an improved understanding of lead solder corrosion control. Here, we assessed zinc orthophosphate’s ability to inhibit nitrate-accelerated lead solder corrosion at the affected utility using complementary bench-scale tests with new lead–tin solder, harvested pipe samples, and intensive field monitoring in consumers’ homes. The hypothesized mechanism of zinc orthophosphate in controlling galvanic lead solder corrosion was examined as part of this evaluation.

## Materials and Methods

### Utility Field Data

In 2017, the utility switched from high alkalinity (340 mg/L CaCO_3_), low CSMR (0.14), pH 7.8 groundwater with nondetectable nitrate to pH 7.5 surface water with lower alkalinity (31 mg/L CaCO_3_), higher CSMR (0.53), pH of 7.5, and variable nitrate. Beginning in May 2019, monthly first draw LCR compliance samples were obtained from 26 to 72 homes with leaded solder in the affected midwestern community with no known lead service lines.^23^ All lead samples were acidified with 2% HNO_3_ and digested for at least 24 h before being analyzed with inductively coupled plasma mass spectrometry (ICP-MS).

### Lead Solder Coupon Study

Coupons were prepared by melting 1 ± 0.05 g of new 50:50 lead–tin solder along the interior of 1-in length and 3/8-in diameter copper coupling. Coupons were placed in 125 mL glass jars and conditioned for a week in the original groundwater used by the utility before being exposed to the new surface water to replicate the source water change. Surface water was shipped weekly by the utility and augmented to create a total of 15 water conditions that were adjusted throughout the course of the experiment (Table 1). Phase 1 was designed to compare the inhibitory effects of zinc and orthophosphate, separately and in combination, for solder exposed to nitrate (*n* = 15). An orthophosphate dose of 1 mg/L as P and a 3:1 ratio of orthophosphate to zinc were selected for the zinc orthophosphate condition based on successful performance in previous case studies.^24^ In Phase 2, the Phase 1 test groups were further subdivided, and nitrate levels were varied to explore the possibility of a threshold effect (*n* = 5). During Phase 3, these nitrate levels were increased for select groups to explore the effect of even higher nitrate levels. Phases 4 and 5 explored the inhibitory effects of varying the ratio of zinc to phosphate. Water in the jars was changed three times per week using a static dump-and-fill protocol (weekly composite samples were collected for each individual coupon) and analyzed with ICP-MS following a 24 h minimum digestion with 2% HNO_3_. Averages for each group of samples were then calculated from the concentration in each composite sample.

**Table 1:** Summary of Amendments Made to the Shipped Source Water throughout the 404 Day Study for the 15 Groups of Coupons Tested^a^^,^^1^

Condition	Phase 1 (Day 0–88)	Phase 2 (Day 89–123)	Phase 3 (Day 124–172)	Phase 4 (Day 173–214)	Phase 5 (Day 215–404)
A	Control	Control	Control	Control	Control
B	+5 mg/L NO_3_	+1 mg/L NO_3_	+1 mg/L NO_3_	+1 mg/L NO_3_	-
C	+5 mg/L NO_3_	+3 mg/L NO_3_	+3 mg/L NO_3_	+3 mg/L NO_3_	-
D	+5 mg/L NO_3_	+5 mg/L NO_3_	+8 mg/L NO_3_	+8 mg/L NO_3_	+8 mg/L NO_3_
E	Control	+1 mg/L P	+1 mg/L P	+1 mg/L P	+1 mg/L P
F	+1 mg/L P	+1 mg/L P	+1 mg/L P	+0.66 mg/L Zn	-
	+5 mg/L NO_3_	+1 mg/L NO_3_	+1 mg/L NO_3_	+1 mg/L P	
				+1 mg/L NO_3_	
G	Control	+1 mg/L P	+1 mg/L P	+1 mg/L P	+1 mg/L P
		+2 mg/L NO3	+7 mg/L NO3	+7 mg/L NO3	+8 mg/L NO3
H	+1 mg/L P	+1 mg/L P	+1 mg/L P	+0.66 mg/L Zn	-
	+5 mg/L NO_3_	+3 mg/L NO_3_	+3 mg/L NO_3_	+1 mg/L P	
				+3 mg/L NO_3_	
I	+1 mg/L P	+1 mg/L P	+1 mg/L P	+0.66 mg/L Zn	+0.66 mg/L Zn
	+5 mg/L NO_3_	+5 mg/L NO_3_	+8 mg/L NO_3_	+1 mg/L P	+1 mg/L P
				+8 mg/L NO_3_	+8 mg/L NO_3_
J	+0.33 mg/L Zn	+0.33 mg/L Zn	+0.33 mg/L Zn	+0.33 mg/L Zn	+0.33 mg/L Zn
	+1 mg/L P	+1 mg/L P	+1 mg/L P	+1 mg/L P	+1 mg/L P
	+5 mg/L NO_3_	+1 mg/L NO_3_	+1 mg/L NO_3_	+1 mg/L NO_3_	
K	+0.33 mg/L Zn	+0.33 mg/L Zn	+0.33 mg/L Zn	+0.33 mg/L Zn	+0.16 mg/L Zn
	+1 mg/L P	+1 mg/L P	+1 mg/L P	+1 mg/L P	+1 mg/L P
	+5 mg/L NO_3_	+3 mg/L NO_3_	+3 mg/L NO_3_	+3 mg/L NO_3_	+8 mg/L NO_3_
L	+0.33 mg/L Zn	+0.33 mg/L Zn	+0.33 mg/L Zn	+0.33 mg/L Zn	+0.33 mg/L Zn
	+1 mg/L P	+1 mg/L P	+1 mg/L P	+1 mg/L P	+1 mg/L P
	+5 mg/L NO_3_	+5 mg/L NO_3_	+8 mg/L NO_3_	+8 mg/L NO_3_	+8 mg/L NO_3_
M	+0.33 mg/L Zn	+0.33 mg/L Zn	+0.33 mg/L Zn	+0.33 mg/L Zn	-
	+5 mg/L NO_3_	+1 mg/L NO_3_	+1 mg/L NO_3_	+1 mg/L NO_3_	
N	+0.33 mg/L Zn	+0.33 mg/L Zn	+0.33 mg/L Zn	+0.33 mg/L Zn	-
	+5 mg/L NO_3_	+3 mg/L NO_3_	+3 mg/L NO_3_	+3 mg/L NO_3_	
O	+0.33 mg/L Zn	+0.33 mg/L Zn	+0.33 mg/L Zn	+0.33 mg/L Zn	-
	+5 mg/L NO_3_	+5 mg/L NO_3_	+8 mg/L NO_3_	+8 mg/L NO_3_	

a Control indicates no amendments made to source water, all other cases describe the amount of zinc, nitrate, and phosphate added to the source water. In each phase, coupons that received zinc were first conditioned with 4 mg/L Zn for 5 days to expedite scale formation before receiving the dose shown below. All nitrate values are in mg/L NO_3_–N. Experimental results for conditions A–I during the first 172 days were previously reported.

### Harvested Pipe Study

Thirteen copper pipes were extracted from a home that consistently had the highest lead levels in the field sampling pool. The pipes were removed in May 2021, after being exposed to the groundwater for approximately 42 years and the new surface water source for over 4 years in situ. To replicate the effect of the source water change, the pipes were capped and filled with water and conditioned with the original groundwater for a week (Phase 1) before switching to the surface water source. The pipes were then divided into two groups based on whether they had visible exterior solder joints (*n* = 5) or not (*n* = 8). All pipes received 1 mg/L orthophosphate as P and additional zinc and nitrate treatments as described below (Table 2). During Phase 2, half of the pipes with and without visible solder were treated with nitrate to explore the relationship between visible exterior solder and lead release. In Phase 3, zinc was added to the pipe releasing the most lead at a 1:3 ratio with orthophosphate. Zinc was added to half of the remaining pipes, including some treated with and without nitrate, in Phase 4. Water changes occurred three times per week using a static dump-and-fill protocol and weekly composite samples were collected for each individual pipe and analyzed in the same manner as the coupons. In some cases, 20% HNO_3_ and heating at 50 °C for 24 h were needed to facilitate digestion of large particulates containing tin.^25^

**Table 2:** Summary of Amendments Made to the Shipped Source Water throughout the 392 Day Study for the 13 Harvested Pipes Tested^a^

Condition	Pipe Type	*n*	Phase 1 (Day 0–48)	Phase 2 (Day 49–97)	Phase 3 (Day 98–254)	Phase 4 (Day 255–392)
A	No visible solder	2				
B	No visible solder	2		+7.4 mg/L NO_3_	+7.4 mg/L NO_3_	+7.4 mg/L NO_3_
C	No visible solder	2				+0.33 mg/L Zn
D	No visible solder	2		+7.4 mg/L NO_3_	+7.4 mg/L NO_3_	+7.4 mg/L NO_3_
						+0.33 mg/L Zn
E	Visible solder	2				
F	Visible solder	2		+7.4 mg/L NO_3_	+7.4 mg/L NO_3_	+7.4 mg/L NO_3_
G	Visible solder	1		+7.4 mg/L NO_3_	+7.4 mg/L NO_3_	+7.4 mg/L NO_3_
					+0.33 mg/L Zn	+0.33 mg/L Zn

a Each pipe received 1 mg/L orthophosphate as P and were supplemented with zinc and nitrate treatments listed below. Pipes that received zinc were first conditioned with 4 mg/L Zn for 5 days to expedite scale formation before receiving the dose shown below. Experimental results for all conditions through the first 98 days were previously reported.^1^ All nitrate values are in mg/L NO_3_–N.

### SEM Analysis

Coupon and pipe segments were analyzed for visual signs of corrosion using an FEI Quanta 600 environmental scanning electron microscope (ESEM). The accelerating voltage was set to 30 kV. Energy-dispersive spectroscopy (EDS) analysis was used to characterize the chemical composition of the pipe scale, and the limit of detection was 0.1%. Measurements below the detection limit were reported as half the limit.

### Statistical Analysis

Statistical analysis for the three studies included linear regressions and pooled ANOVAs using lead, nitrate, and CSMR as parameters. All analyses were conducted in R (version 4.1.1) using an alpha (α) value of 0.05.

## Results

### Lead Solder Coupon Study

#### Phase 1 (Days 0–88): Zinc Orthophosphate Offered Immediate Control of Nitrate-Accelerated Lead Solder Corrosion

**Figure 1:**
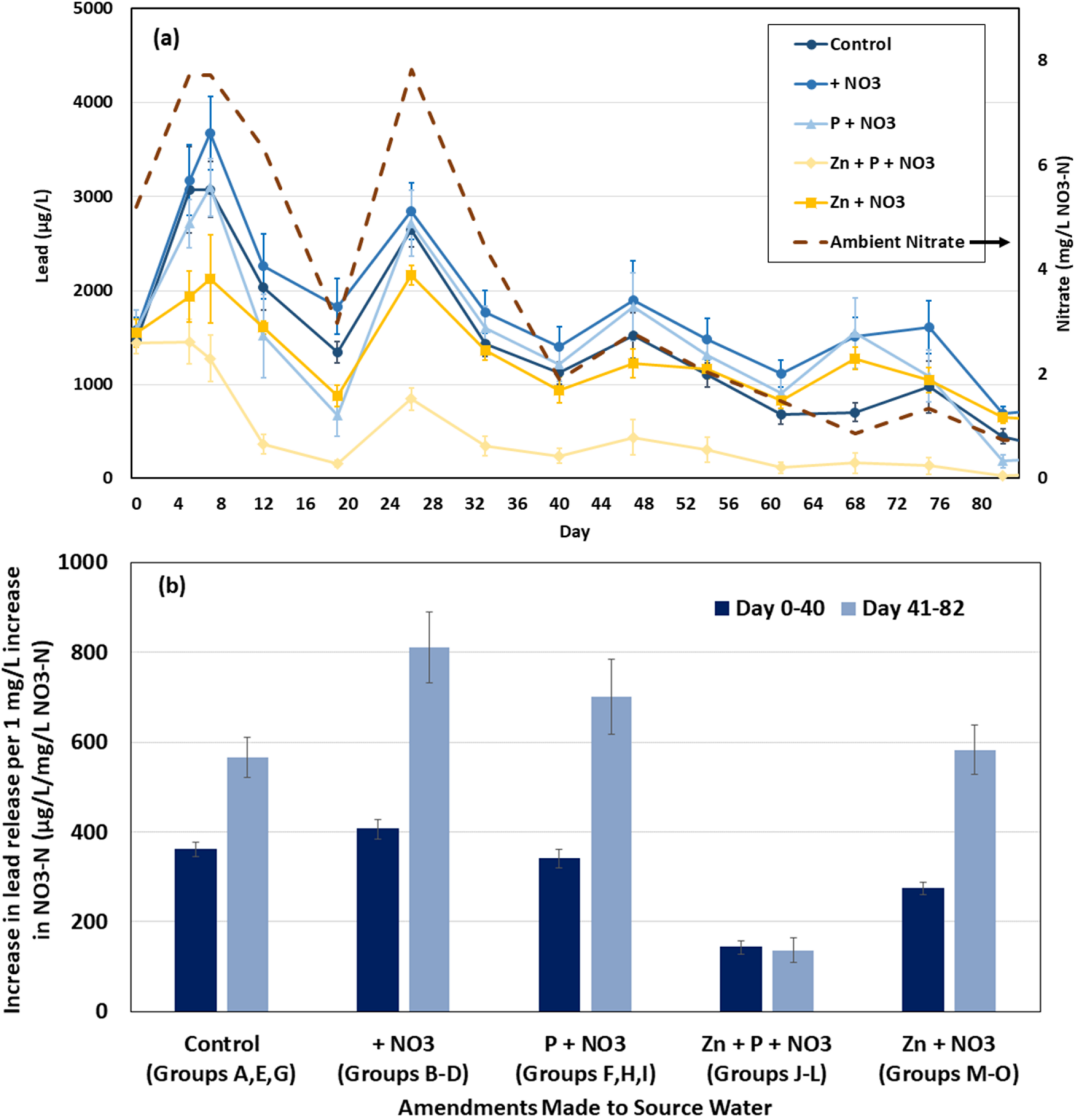
(a) Lead release and ambient surface water nitrate versus time during Phase 1 of the coupon study. Control condition includes coupon groups A, E, G; + NO_3_ condition includes coupon groups B–D; P + NO_3_ condition includes coupon groups F, H, I; Zn + P + NO_3_ condition includes coupon groups J–L; and Zn + NO_3_ condition includes coupon groups M–O (Table 4.1). Error bars represent 95% confidence intervals (*n* = 15). Adapted from “Seasonal fluctuations in nitrate levels can trigger lead solder corrosion problems in drinking water,” by ref (1). Copyright 2023 American Chemical Society. (b) Increase in lead release per 1 mg/L increase in nitrate (slopes) from regression models using lead release and ambient source water nitrate for each of the five conditions (*n* = 15) during the first and second half of the Phase 1 study. Error bars represent 95% confidence intervals.

Following the conditioning phase and prior to treatment, all coupon groups (*n* = 15) began the study with an average lead release of 1530 ± 91 μg/L. During the first 7 days of the experiment, runoff following a heavy rain event caused ambient nitrate levels in the shipped surface water to reach 7.7 mg/L NO_3_–N and the CSMR to reach 0.79 (Figures 1a and S1, and measurement ranges shown in Figure S2). In response to this natural variation, average lead release during that time increased by 37–133% for all conditions except those treated with zinc orthophosphate. Average lead release increased in the following order: augmented nitrate condition with zinc alone (2130 μg/L, range: 1360–4880 μg/L), control condition without additional nitrate (3070 μg/L, range: 2240–3960 μg/L), augmented nitrate condition with phosphate (3100 μg/L, range: 2030–3950 μg/L), and augmented nitrate condition without an inhibitor (3670 μg/L, range: 2530–4900 μg/L). In marked contrast, lead release from the coupons treated with zinc orthophosphate decreased to 1280 μg/L (range: 704–2360 μg/L). Despite the fluctuations in water chemistry throughout Phase 1, zinc orthophosphate offered a significant advantage in lead control compared to either orthophosphate or zinc alone (*p* = 1.7 × 10^–3^ to 0.016, ANOVA with F(4, 70) = 7.7, effect size η^2^ = 0.34) and by day 82 average lead levels dropped to 30 μg/L (a 95–99% reduction in lead release).

For most conditions, lead release was an approximately linear function of nitrate in the experimental water. Linear regression analysis using lead release paired with ambient source water nitrate during days 0–40 and 41–82 showed that every 1 mg/L increase in nitrate was associated with an increase in lead of 137–143 μg/L for zinc orthophosphate, 341–701 μg/L for orthophosphate, 275–582 μg/L for zinc, and 406–811 μg/L for the augmented nitrate condition without an inhibitor (*r*^2^ = 0.83–0.97, Figures 1b, S2 and Table S1). Moreover, for all conditions except zinc orthophosphate, lead release per 1 mg/L increase in nitrate increased between days 0–40 and 41–82 as the solder degraded and began to spall off in large pieces visible to the naked eye.

#### Phases 2 and 3 (Days 89–172): The Addition of Zinc Dampened the Correlation between Lead Release and Varying Doses of Nitrate

**Figure 2:**
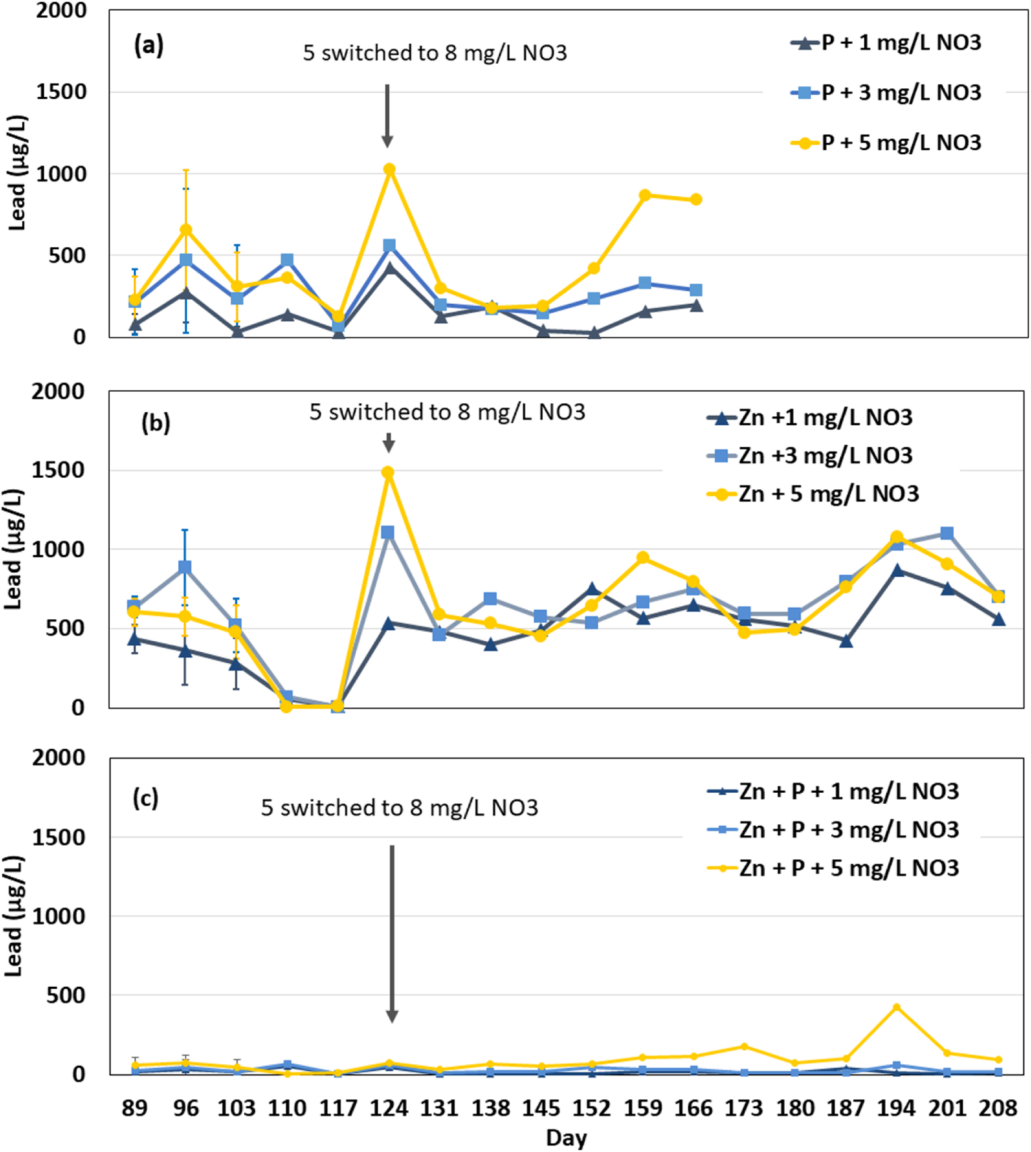
Average lead release during Phases 2–4 from coupons augmented with 1, 3, or 5 mg/L NO_3_–N beyond ambient source water nitrate and (a) orthophosphate (Groups F, H, and I), (b) zinc alone (Groups M–O), and (c) zinc orthophosphate (Groups J–L). Coupons receiving 5 mg/L began receiving 8 mg/L NO_3_–N on day 124. Error bars represent 95% confidence intervals. After day 103, composite samples were collected (*n* = 5).

On day 89, the five original coupon groups were divided into subgroups (*n* = 5) to examine the effect of treatment with 1, 3, or 5 mg/L NO_3_–N on inhibitor performance. As mentioned in a prior work, orthophosphate generally reduced lead release compared to the augmented nitrate condition without an inhibitor.^1^ At any given time during Phase 2, lead release with orthophosphate was 33–70% lower than the augmented nitrate condition without an inhibitor when supplemented nitrate was at or above 3 mg/L NO_3_–N. At this point in the study, lead release did not differ significantly between coupons treated with orthophosphate and the addition of 1, 3, or 5 mg/L NO_3_–N (*p* = 0.151–0.953, ANOVA, Figures 2a and S4a). However, when nitrate treatment was increased from 5 to 8 mg/L NO_3_–N at the beginning of Phase 3, average lead release for the orthophosphate condition increased from 337 to 537 μg/L compared to the corresponding control that increased only from 800 to 836 (Table S2). There was only one instance where lead release was 7% higher with orthophosphate than without when 8 mg/L NO_3_–N was added.

Treatment with zinc alone also offered little ability to control nitrate-accelerated lead solder corrosion and during Phase 2 only reduced lead release by up to 41% compared to the augmented nitrate condition without an inhibitor when supplemented nitrate was at or above 3 mg/L NO_3_–N. There were multiple times when lead release was higher with zinc (up to 14%) than without. Nitrate treatment significantly impacted lead release from the coupons treated with zinc, and the addition of 3 mg/L NO_3_–N was as corrosive as 5 mg/L NO_3_–N (*p* = 4.83 × 10^–5^ to 0.39, ANOVA, Figures 2b and S4b). When nitrate for the zinc-treated coupons exposed to 5 mg/L NO_3_–N was increased to 8 mg/L NO_3_–N in Phase 3, average lead release from that condition increased from 614 to 778 μg/L. Clearly, zinc alone does not control lead release even in the presence of lower nitrate levels.

In contrast, zinc orthophosphate treatment produced the lowest levels of lead during Phase 2 when nitrate treatment was at or above 3 mg/L, causing a reduction of 87–99% lead release in comparison to the augmented nitrate condition without an inhibitor (*p* = 1 × 10^–7^ to 0.06, Figures 2c and S4c). Thus, the combination of zinc and orthophosphate had a synergistic effect for control of nitrate-accelerated lead solder corrosion. Additionally, the coupons treated with zinc orthophosphate did not release significantly different levels of lead during this phase regardless of whether they were treated with 1,3, or 5 mg/L NO_3_–N (*p* = 0.08–0.85, ANOVA). When nitrate treatment for the zinc orthophosphate-treated coupons receiving 5 mg/L NO_3_–N was increased to 8 mg/L NO_3_–N, average lead release only increased from 39 to 74 μg/L—a much smaller increase than was observed for other conditions.

Similar trends in inhibitor performance were observed for tin and copper. As may be expected for a 50:50 lead–tin solder alloy, trends in tin corrosion control resembled those of lead, with nitrate exacerbating corrosion and zinc orthophosphate offering the most inhibition (Figure S5a–c). Increased corrosion of the sacrificial anode enhances cathode protection, and while higher nitrate levels caused higher rates of lead and tin corrosion, higher nitrate levels generally caused less copper release from the coupons treated with orthophosphate or zinc alone (*p* = 1 × 10^–7^ to 0.713, Figure S6a–b). However, in the presence of zinc orthophosphate, copper was not correlated to nitrate (*p* = 0.57–0.99, Figure S6c).

#### Phases 4 and 5 (Days 173–404): Short-Term Effect of Higher Zinc Levels

In Phase 4, the orthophosphate coupons treated with 1, 3, or 8 mg/L NO_3_–N began receiving twice as much zinc (0.66 mg/L Zn) as had been applied to the zinc orthophosphate coupons. Time was an important factor in zinc orthophosphate’s ability to reduce lead levels after switching from orthophosphate. After 42 days of treatment, average lead release from these coupons dropped from 168–547 μg/L to 94–342 μg/L (Table 3). While coupons treated with 1 and 3 mg/L NO_3_–N were removed from the study on day 222, those treated with 8 mg/L NO_3_–N continued to receive the higher zinc orthophosphate dose through day 404. Average lead release further declined to 194 μg/L in Phase 5 (−1.28 μg/L/day in Phase 5) and after 2.5 months, these coupons were not statistically different than coupons that had been treated with the original zinc orthophosphate dose from the start of the experiment (*p* = 0.41, ANOVA, Figure S7).

**Table 3:** Averages and Ranges of Lead Release from Coupons Originally Treated with Orthophosphate (Groups F, H, and I) and Zinc Orthophosphate (Groups J–L) in Phases 3–5^a^

Phase 3 (Day 124–172)					Phase 4 (Day 173–214)			Phase 5 (Day 215–404)		
Condition	Average Lead (μg/L)		Range (μg/L)	Condition		Average Lead (μg/L)	Range (μg/L)	Condition	Average Lead (μg/L)	Range (μg/L)
P +1 mg/L NO_3_	168	30.1–428			0.66 mg/L Zn + P +1 NO_3_	132	10.1–354	-	-	-
P +3 mg/L NO_3_	277	149–558			0.66 mg/L Zn + P +3 NO_3_	94	16.8–240	-	-	-
P +8 mg/L NO_3_	547	178–1020			0.66 mg/L Zn + P +8 NO_3_	342	103–805	0.66 mg/L Zn + P +8 NO_3_	194	30.8–650
0.33 mg/L Zn + P +1 NO_3_	17.2	5.8–13.1			0.33 mg/L Zn + P +1 NO_3_	15.8	7.2–40.5	0.33 mg/L Zn + P	15.5	3.1–41.2
0.33 mg/L Zn + P +3 NO_3_	32	13.1–33.0			0.33 mg/L Zn + P +3 NO_3_	23.6	9.9–60.5	0.16 mg/L Zn + P +8 NO_3_	211	14.0–716
0.33 mg/L Zn + P +8 NO_3_	74.2	33.0–113			0.33 mg/L Zn + P +8 NO_3_	168	73.4-428	0.33 mg/L Zn + P +8 NO_3_	129	17.8–538

a Changes to treatment conditions for each phase are noted accordingly. Two of the phosphate groups (Groups F and H) were removed from the study on day 222, as indicated by the gray boxes.

While an increase in zinc helped reduce lead release, a decrease in zinc could also increase lead levels. In Phase 5, coupons treated with zinc orthophosphate and 3 mg/L NO_3_–N began to receive half the original dose of zinc (0.16 mg/L Zn) and saw a gradual increase in lead levels from 24 μg/L on average in Phase 4 to 211 μg/L on average in Phase 5 (0.127 μg/L/day). Water utilities will need to weigh the benefits of adding zinc on lead control in the distribution system with its potential adverse impacts on wastewater facilities where zinc is a regulated contaminant.

### Harvested Pipe Study

#### Zinc Orthophosphate Reduced Lead Release from Decades-Old Solder by an Order of Magnitude, But after a Period of Months, Periodic Lead Spikes Still Occurred

**Figure 3:**
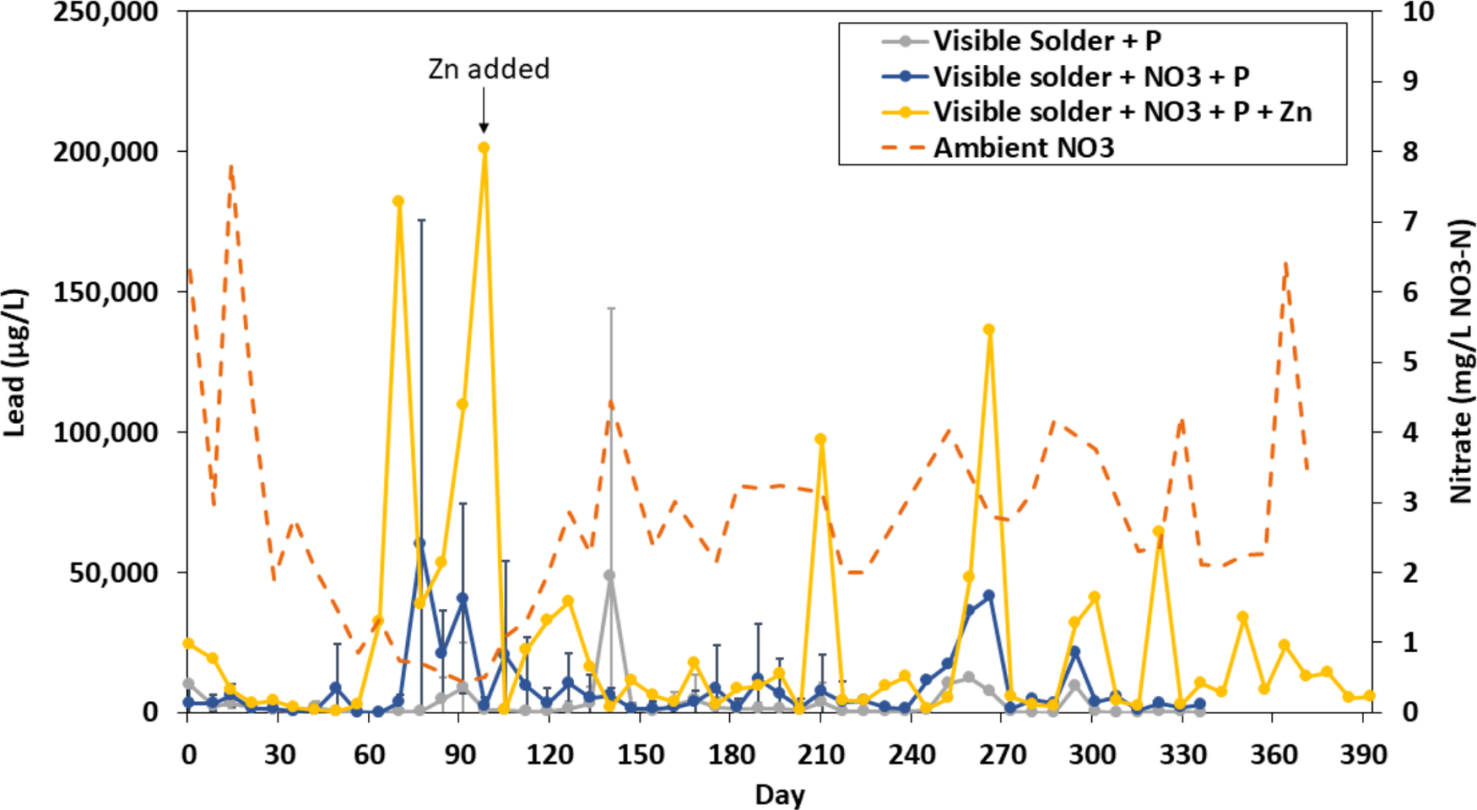
Lead release from the harvested pipes with visible solder (Pipes E, F, and G) and ambient nitrate levels versus time. Nitrate was added to select pipes (Pipes F and G) on day 56 following a conditioning phase. Zinc orthophosphate was added to one pipe (Pipe G) on day 98. All pipes received 1 mg/L orthophosphate as P. Error bars represent 95% confidence intervals. Adapted from “Seasonal fluctuations in nitrate levels can trigger lead solder corrosion problems in drinking water,” by ref (1). Copyright 2023 American Chemical Society.

**Figure 4:**
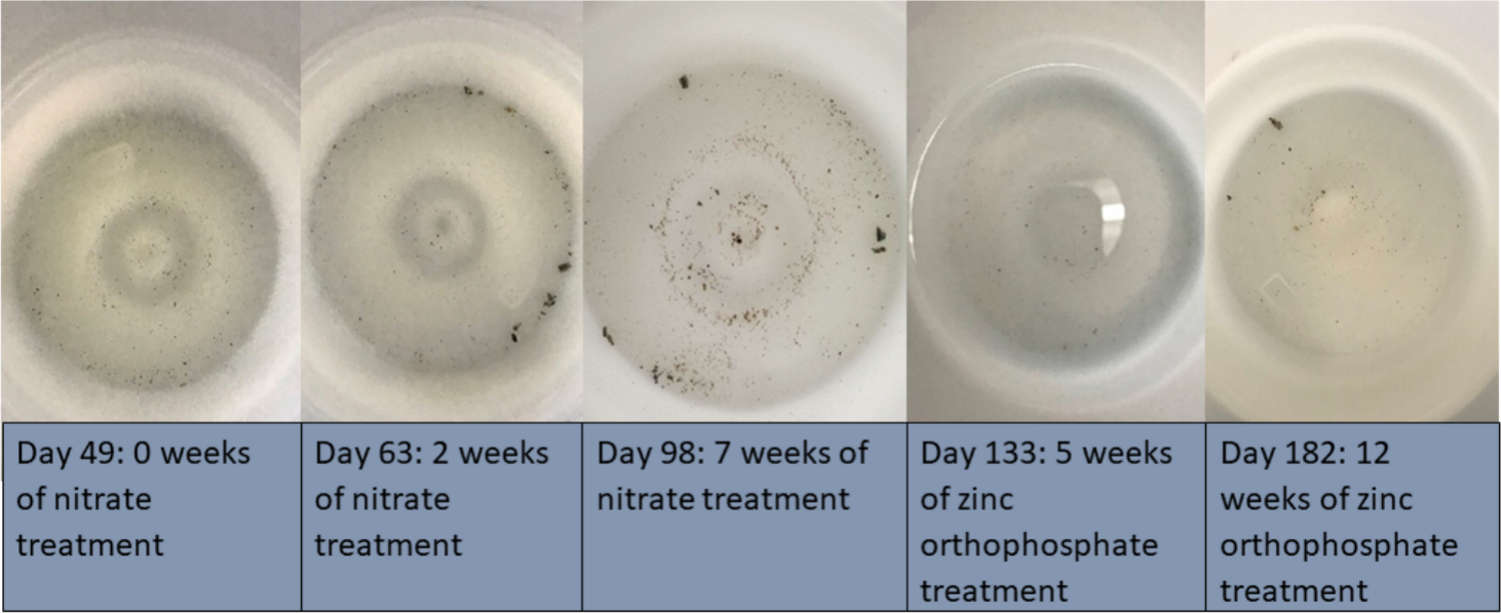
Particulate release from Pipe G over time, before and after the addition of zinc orthophosphate. Pictures are a top-down view of the weekly collection container.

After 56 days of conditioning in the surface water with orthophosphate (Phase 1), subsets of the harvested pipes were suddenly dosed with 7.4 mg/L NO_3_–N to simulate a nitrate spike (Phase 2). Ambient nitrate in the surface water was <1.5 mg/L NO_3_–N, while the CSMR was about 0.50 during this time. As described in a prior publication, the pipes with visible exterior solder that received the augmented nitrate treatment saw an increase in lead release by 1–3 orders of magnitude (Figures 3,and S8) and large solder particle spallation began shortly thereafter.^1^ Of those three pipes, Pipe G (which consistently released the most lead) began receiving 0.33 mg/L Zn on day 98 (Phase 3) and saw a dramatic reduction in lead levels from 103,000 μg/L on average between days 63 and 98 down to 18,700 μg/L on average between days 105 and 392 (an 82% reduction in lead release). Following the addition of zinc, particle spallation from Pipe G also decreased (Figure 4). While zinc orthophosphate appeared to reduce lead release, lead levels never returned to their original values before nitrate spike on day 56 and there were still spikes in lead release. Pipes without visible solder did not show the same fluctuations in lead release when nitrate was added (Figure S9).

#### Iron Deposits May Impede Zinc Orthophosphate’s Ability to form a Protective Scale

Throughout the study, zinc recovery in the effluent of the harvested pipe with visible solder receiving zinc orthophosphate treatment (Pipe G) was generally very low (median = 8.8%), despite an initial conditioning dose of 4 mg/L Zn. This was in contrast with the new solder coupon study, wherein zinc was fully recovered in the effluent of the coupons early on and zinc orthophosphate exhibited more than a 90% reduction in lead levels compared to the control conditions. Pipe G was later found to have a significant coating of iron rust. In order to address whether the presence of iron could affect zinc orthophosphate’s ability to form a protective scale, zinc was applied to half of the pipes with no visible exterior solder (Pipe Groups C and D) on day 252 (Phase 4). These pipes had also high levels of iron in their effluent (median of 876 μg/L). Even at the end of the study, no more than 7% of applied zinc was recovered in the effluent of these pipes and Pipe G. It was speculated that visible iron deposits in these harvested pipes were sorbing the zinc. Some distribution system sampling of water mains also confirmed increasing levels of zinc in water over a period of 6 months, presumably as the scale of the old pipes in the system was slowly coated with applied zinc (Figure S10). The average pH in the distribution system when surface water was used was 7.5, which is within the pH range that iron hydroxide is known to sorb zinc.^26,27^

### Utility Field Data

#### Zinc Orthophosphate Reduced 90th Percentile Lead Levels in the Drinking Water System

**Figure 5:**
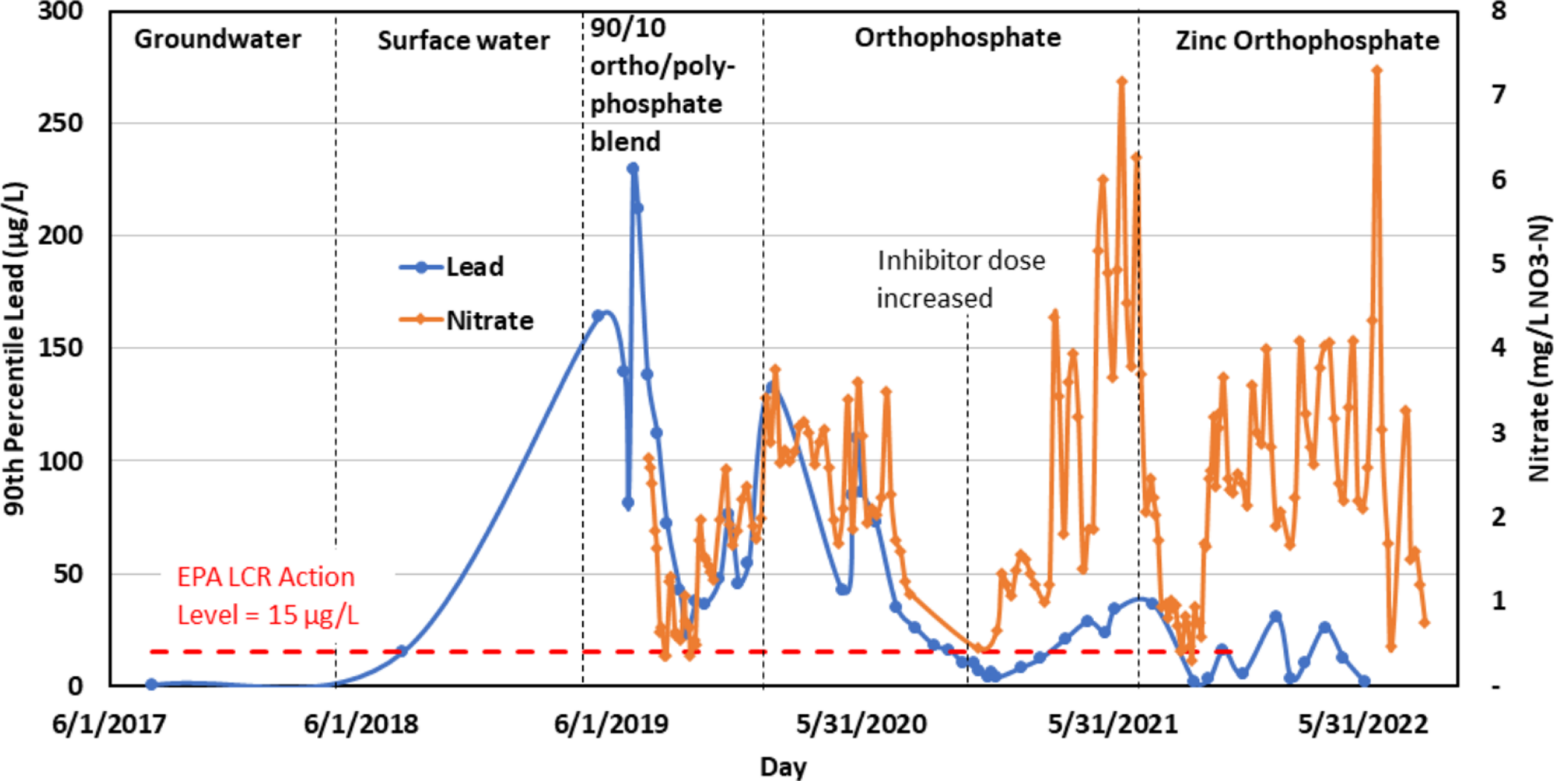
90th percentile lead release and fluctuations in source water nitrate levels over time in the affected case study community. Changes in corrosion control are noted and the U.S. EPA Lead and Copper Rule Action Level of 15 μg/L is indicated by the red line. Adapted from “Seasonal fluctuations in nitrate levels can trigger lead solder corrosion problems in drinking water,” by ref (1). Copyright 2023 American Chemical Society.

After observing the promising bench-scale coupon and harvested pipe results with zinc orthophosphate treatment, the utility in the case study began applying zinc orthophosphate (0.33 mg/L Zn and 1 mg/L P) in August 2021. Throughout this treatment period, nitrate levels ranged from 0.3 to 7.3 mg/L NO_3_–N (2.21 mg/L on average) and CSMR ranged from 0.26 to 0.84 (0.52 on average). Lead levels remained consistently below 30 μg/L (11 μg/L on average) even as nitrate and CSMR levels fluctuated (Figures 5 and S11). Only 3 out of 10 sampling events (30%) had a 90th percentile lead value above 15 μg/L with zinc orthophosphate treatment compared to 14 out of 22 sampling events (64%) with orthophosphate treatment. As was true for the bench-scale studies, zinc orthophosphate appeared to reduce but did not completely prevent lead release. Multiple regression analysis using source water nitrate data paired with 90th percentile lead data indicated that zinc orthophosphate offered further corrosion control beyond that of orthophosphate. Specifically, after spallation became problematic, there was no correlation between nitrate and lead using orthophosphate (*p* = 0.70, *r*^2^ = 0.65), but there was still a relationship with CSMR (*p* = 1.4 × 10^–3^). In contrast, nitrate was not significantly correlated with lead (*p* = 0.35, *r*^2^ = 0.15) nor CSMR (*p* = 0.56, Table 4) when zinc orthophosphate was applied. The considerable weakening of the *r*^2^ term and the general reduction in lead levels point to zinc orthophosphate’s efficacy as a corrosion inhibitor for lead solder in water containing higher nitrate levels. Trends in lead release suggest that zinc orthophosphate’s performance further improved with time. By December 2022, 90th percentile lead dropped down to 7.2 μg/L and in July 2024, almost three years after the initial application of zinc orthophosphate, 90th percentile lead was 1.5 μg/L.

**Table 4:** Results from Multiple Regression Analysis Using Utility-Supplied Source Water Nitrate Data and 90th Percentile Lead Values^a^

Condition	*r*^2^	Intercept	Intercept *p*-value	NO_3_ Slope (μg/L Pb/mg/L NO_3_–N)	NO_3_*p*-value	CSMR Slope (μg/L Pb/CSMR)	CSMR *p*-value
90/10 Ortho/Poly phosphate Blend	0.79	–152	0.13	34	3.4 × 10^–3^	366	0.12
Orthophosphate	0.65	–122	5.3 × 10^–3^	–2	0.70	334	1.4 × 10^–3^
	0.15	15	0.59	6	0.35	–37	0.56

a Data was broken into phases where different corrosion inhibitors were applied. Slopes correspond to changes in lead release per 1 unit change in nitrate or CSMR.

### Insights into Zinc Orthophosphate’s Corrosion Inhibition Mechanism

#### SEM Analysis of the Harvested Pipe Surface Showed that Zinc and Orthophosphate Codeposit at the Copper–Solder Interface

**Figure 6:**
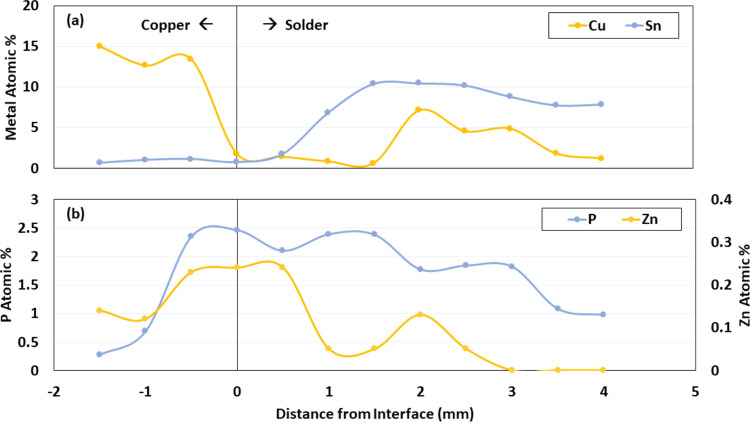
(a) Atomic percent of cathodic copper and anodic tin near a galvanic interface on a harvested pipe with metallic solder and (b) deposition of zinc and phosphate along the same galvanic interface as determined by SEM.

According to mixed inhibitor theory, galvanic corrosion preferentially draws cationic inhibitors to the higher pH cathode surface where they can inhibit oxygen reduction, whereas anionic inhibitors are concentrated at the lower pH anode surface where they hinder metal oxidation. A synergistic inhibitory effect from reducing both the anodic and cathodic reaction can then be obtained. SEM analysis of pipe scale from a harvested pipe that had been exposed to zinc orthophosphate in the system prior to extraction confirmed that about 0.24% zinc coated the copper cathode at distances >1 mm from the lead–tin solder anode but was undetectable at distances >3 mm from the copper cathode (Figures 6 and S12). Likewise, phosphate was only about 0.3% of the coating on the copper cathode and increased to 1% at distances >3.5 mm from the cathode. All of this is consistent with mixed inhibitor theory.

However, contrary to expectations, the highest levels of both zinc and phosphate occurred at distances within 0.5 mm of the anode–cathode interface. This result suggests a novel mechanism of mixed inhibition that results from the localized formation of a protective zinc orthophosphate scale at the interface where galvanic corrosion is typically most intense, and the solder spallation originates. In retrospect, it is logical that this interface is the point of highest zinc orthophosphate supersaturation because the localized higher pH and concentrated zinc cathodic reaction products mix with the lower pH and concentrated orthophosphate anodic reaction products at this location. Future work should examine this mechanistic hypothesis in greater detail.

## Conclusions

We examined the efficacy of zinc orthophosphate as an inhibitor for nitrate-accelerated lead solder corrosion and determined the following.

- Zinc orthophosphate offered immediate and significant reductions (often more than 90%) in lead release and spallation in bench-scale studies using new lead solder coupons.
- Zinc orthophosphate outperformed orthophosphate or zinc alone in bench-scale studies with new solder, even when nitrate exceeded 7 mg/L NO_3_–N and CSMR remained constant at about 0.54.
- Zinc orthophosphate visibly reduced spallation of lead solder particles due to elevated nitrate in tests with 40 year-old harvested pipes, although some lead spikes could still occur.
- 90th percentile lead in the affected case study community returned below the 15 μg/L lead action level within a year after zinc orthophosphate was added to the system, and less spallation was observed in samples monitored during Lead and Copper Rule testing in consumer homes.
- Zinc orthophosphate’s performance improves with time and is affected by the zinc dose, although impacts on downstream wastewater treatment plants should be considered.
- More than 90% of applied zinc was taken up from stagnant water by the harvested pipes even after 140 days of treatment, suggesting that a higher conditioning dose of zinc orthophosphate may be beneficial in some cases.
- Zinc orthophosphate may control nitrate-accelerated corrosion and spallation by forming a protective scale at the interface between the anode and the cathode, which is typically the location of the most intense galvanic corrosion.

This case study highlights a critical need for additional research on the mechanism of nitrate corrosion of lead solder, how it is affected by water chemistry parameters and cooccurring contaminants, and the effect of other corrosion control strategies such as pH adjustment. Evidence-based updates to corrosion control guidance for waters with higher nitrate due to seasonal runoff or source water changes are also needed.

## Supporting Information Available

This material is available free of charge via the Internet at http://pubs.acs.org.xn--ivg/. Additional experimental details (effect of CSMR, copper and tin release, and SEM). The Supporting Information is available free of charge at https://pubs.acs.org/doi/10.1021/acsestwater.3c00786.

Additional experimental details (effect of CSMR, copper and tin release, and SEM) (PDF)
