# Supplementary material for: Zinc Orthophosphate Can Reduce Nitrate-Induced Corrosion of Lead Solder
Source: ACS ES T Water. 2024 Jul 30;4(8):3153–62. doi: 10.1021/acsestwater.3c00786 (PMC11320579; doi:10.1021/acsestwater.3c00786)
Supplement: Supplementary file 1 — ew3c00786_si_001.pdf [file ew3c00786_si_001.pdf]

SUPPORTING INFORMATION

Zinc Orthophosphate Can Reduce Nitrate-Induced Corrosion of Lead Solder

Kathryn G. Lopez,* Jinghua Xiao, Christopher Crockett, Christian Lytle, Haley Grubbs, and Marc Edwards

AUTHOR INFORMATION:

Corresponding Author

Kathryn G. Lopez – *Department of Civil & Environmental Engineering, Virginia Tech, Blacksburg, VA 24060, United States;* Present Address: ORISE Fellow at US Environmental Protection Agency, Washington, DC 20460, United States

Email: kglopezphd@gmail.com

Authors

Jinghua Xiao – *Aqua America, Bryn Mawr, PA 19010, United States;* Present Address: Circular Water Solution LLC, Ambler, PA 19002, United States

Christopher Crockett – *Aqua America, Bryn Mawr, PA 19010, United States*

Christian Lytle – *Department of Civil & Environmental Engineering, Virginia Tech, Blacksburg, VA 24060, United States*; Present Address: Hazen and Sawyer, Manchester, NH 03104, United States

Haley Grubbs – *Department of Civil & Environmental Engineering, Virginia Tech, Blacksburg, VA 24060, United States*; Present Address: Hazen and Sawyer, Virginia Beach, VA 23462, United States

Marc Edwards – *Department of Civil & Environmental Engineering, Virginia Tech, Blacksburg, VA 24060, United States*

DESCRIPTION OF SUPPORTING INFORMATION:

Additional experimental details (CSMR, copper and tin release, SEM)

TABLE OF CONTENTS:

SI Figure 1. Lead release and CSMR during Phase 1 of coupon study with range error bars

SI Figure 2. Lead release and nitrate during Phase 1 of coupon study with range error bars

SI Figure 3. Linear regressions of lead release versus nitrate during Phase 1 of coupon study

SI Table 1. Summary of linear regression results of lead versus nitrate during coupon study Phase 1

SI Table 2. Lead release data from Phases 2 and 3 of coupon study

SI Figure 4. Lead release during Phases 2 and 4 from coupons treated with inhibitors

SI Figure 5. Tin release during Phases 2 and 4 from coupons treated with inhibitors

SI Figure 6. Copper release during Phases 2 and 4 from coupons treated with inhibitors

SI Figure 7. Average lead release from the 5 phases of the coupon study

SI Figure 8. Lead release from the harvested pipes with visible solder with range error bars

SI Figure 9. Lead release from harvested pipes without visible solder and nitrate levels over time

SI Figure 10. Zinc levels in the distribution system increase over time

SI Figure 11. 90th percentile lead release and fluctuations in CSMR over time in the affected community

SI Figure 12. Region of harvested pipe analyzed with SEM

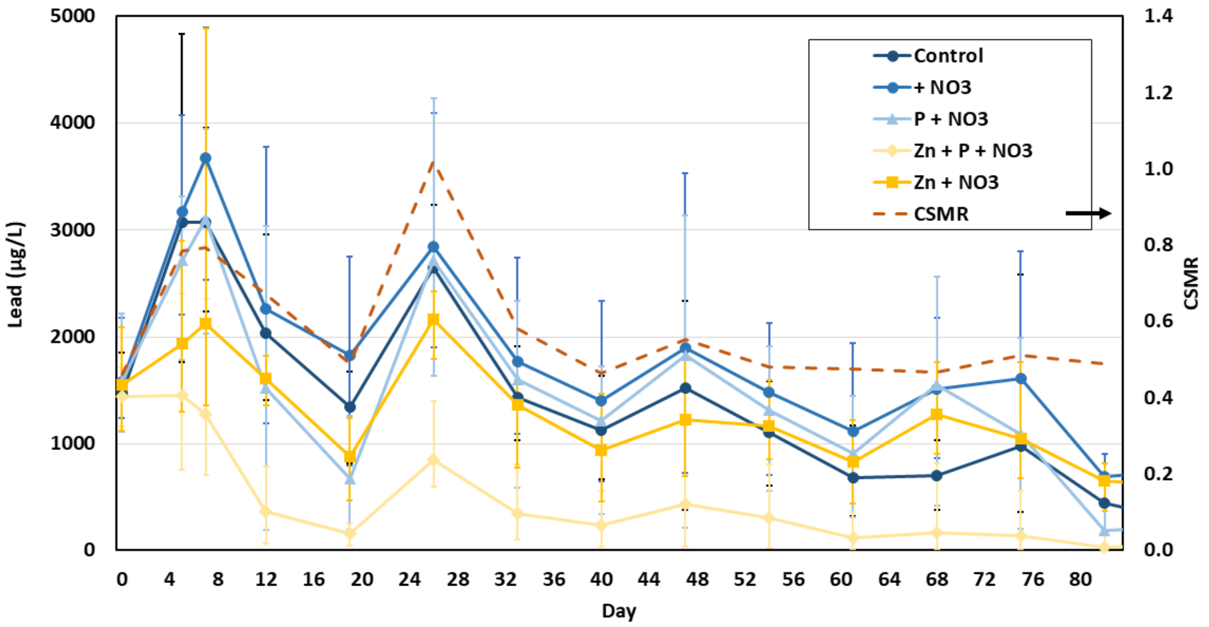

SI Figure 1. Lead release and ambient surface water CSMR versus time during Phase 1 of the coupon study. Error bars represent measurement ranges (n=15). Adapted from "Seasonal fluctuations in nitrate levels can trigger lead solder corrosion problems in drinking water," by Lopez, K. et al., 2023, *Environ. Sci. Technol. Lett.* **10**, p. 21-26. Copyright 2023 American Chemical Society.

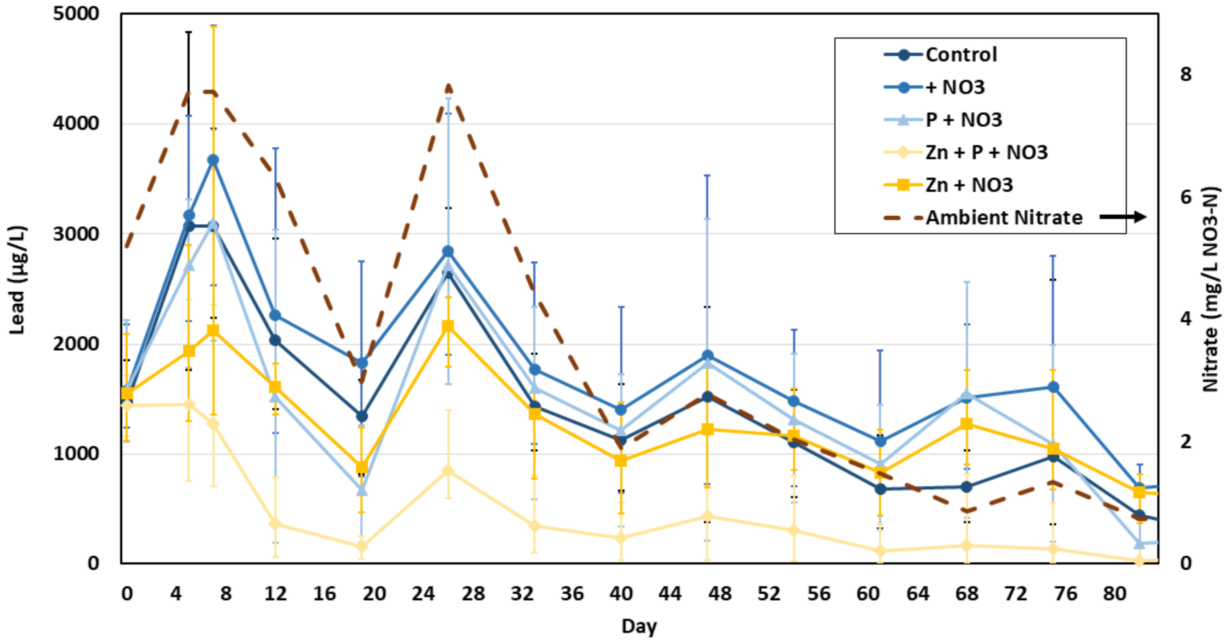

SI Figure 2. Lead release and ambient surface water nitrate versus time during Phase 1 of the coupon study. Error bars represent measurement ranges (n=15). Adapted from "Seasonal fluctuations in nitrate levels can trigger lead solder corrosion problems in drinking water," by Lopez, K. et al., 2023, *Environ. Sci. Technol. Lett.* **10**, p. 21-26. Copyright 2023 American Chemical Society.

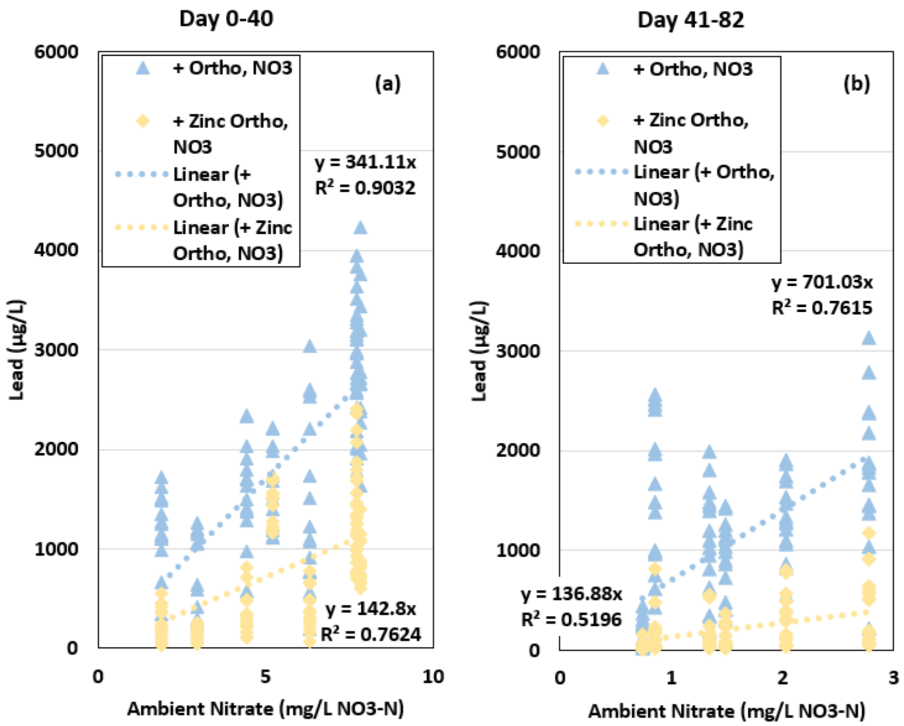

SI Figure 3. Lead release from the coupons versus ambient nitrate in the shipped surface water, along with linear regressions, for the orthophosphate and zinc orthophosphate conditions during a) days 0-40 and b) days 41-82. Data from the linear regressions of these conditions and the rest are provided in SI Table 1.

SI Table 1. Summary of results from the linear regression of nitrate in the source water and lead release from Phase 1 of the coupon study (as demonstrated in SI Figure 3). Slope corresponds to an increase in lead release per unit increase in nitrate.

**Conditions**	**Days 0-40**			**Days 41-82**		
	r^2	**Slope (µg/L Pb/mg/L NO_3-N)**	**NO_3 p value**	r^2	**Slope (µg/L Pb/mg/L NO_3-N)**	**NO_3 p value**
Control	0.940	362	$2.76*10^{-7}$	0.876	566	$2.56*10^{-5}$
+NO_3	0.918	406	$1.87*10^{-6}$	0.822	811	$8.39*10^{-4}$
P + NO_3	0.903	341	$1.87*10^{-6}$	0.762	701	$1.52*10^{-3}$
Zn + P + NO_3	0.762	143	$6.11*10^{-4}$	0.520	137	$3.42*10^{-4}$
Zn +NO_3	0.929	275	$6.30*10^{-8}$	0.833	582	$2.21*10^{-3}$

SI Table 2. Average, minimum, and maximum lead release from coupon Groups D, I, L, and O in Phase 2 and Phase 3 after nitrate treatment was increased from +5 to +8 mg/L NO_3-N.

		**Phase 2 (+5 mg/L NO3)**			**Phase 3 (+8 mg/L NO3)**		
Group	Condition	Average Lead (µg/L)	Min Lead (µg/L)	Max Lead (µg/L)	Average Lead (µg/L)	Min Lead (µg/L)	Max Lead (µg/L)
D	No Inhibitor	800	606	979	836	629	1230
I	Orthophosphate	337	128	656	547	178	1020
L	Zinc Ortho	38.9	5.00	73.2	74.2	33.0	113
O	Zinc	614	476	919	778	454	1480

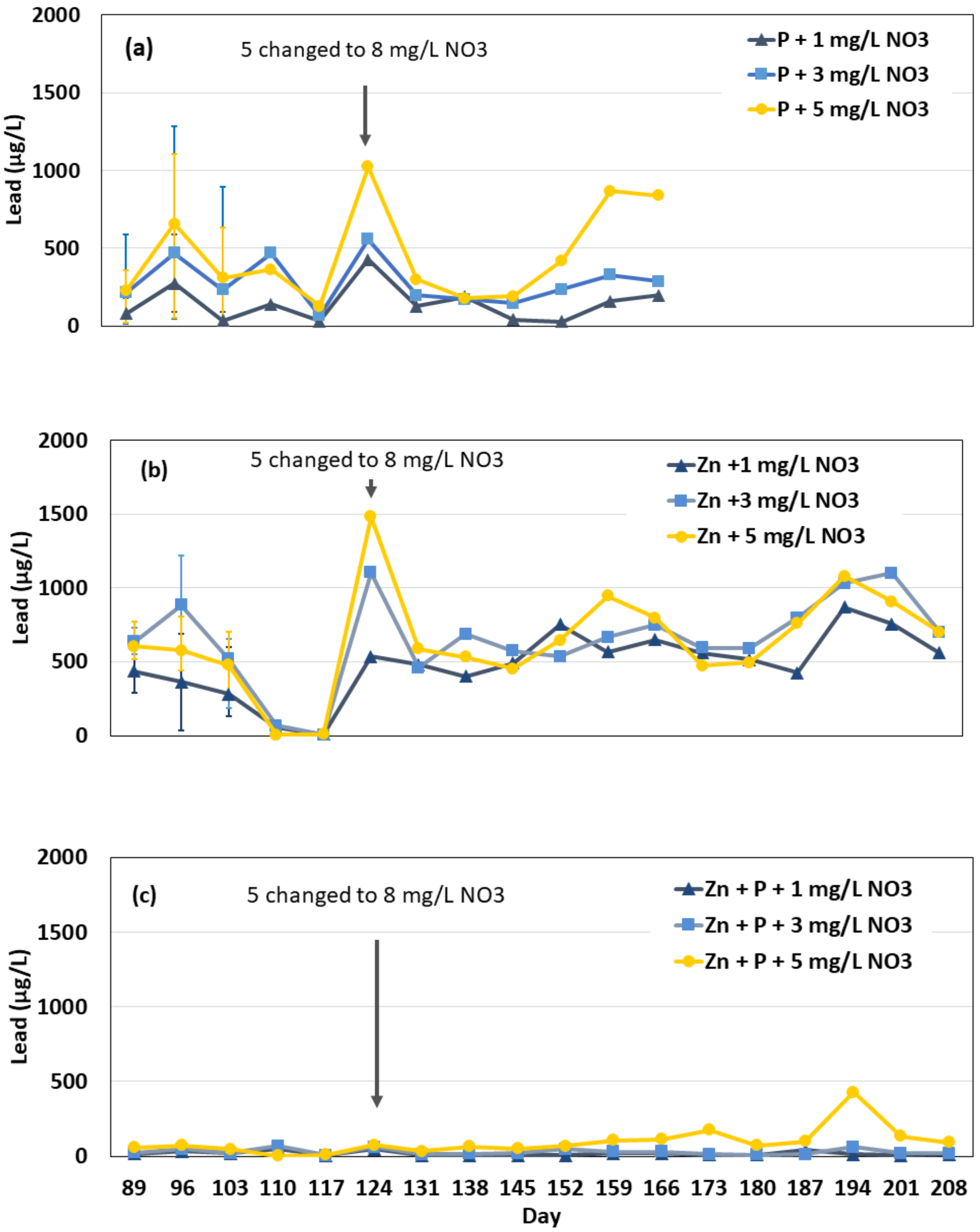

SI Figure 4a-c. Average lead release during Phases 2-4 from coupons augmented with 1, 3, or 5 mg/L NO_3-N beyond ambient source water nitrate and a) orthophosphate (Groups F, H, and I), b) zinc alone (Groups M-O), and c) zinc orthophosphate (Groups J-L). Coupons receiving 5 mg/L began receiving 8 mg/L NO_3-N on day 124. Error bars represent measurement ranges. After day 103, composite samples were collected (n=5).

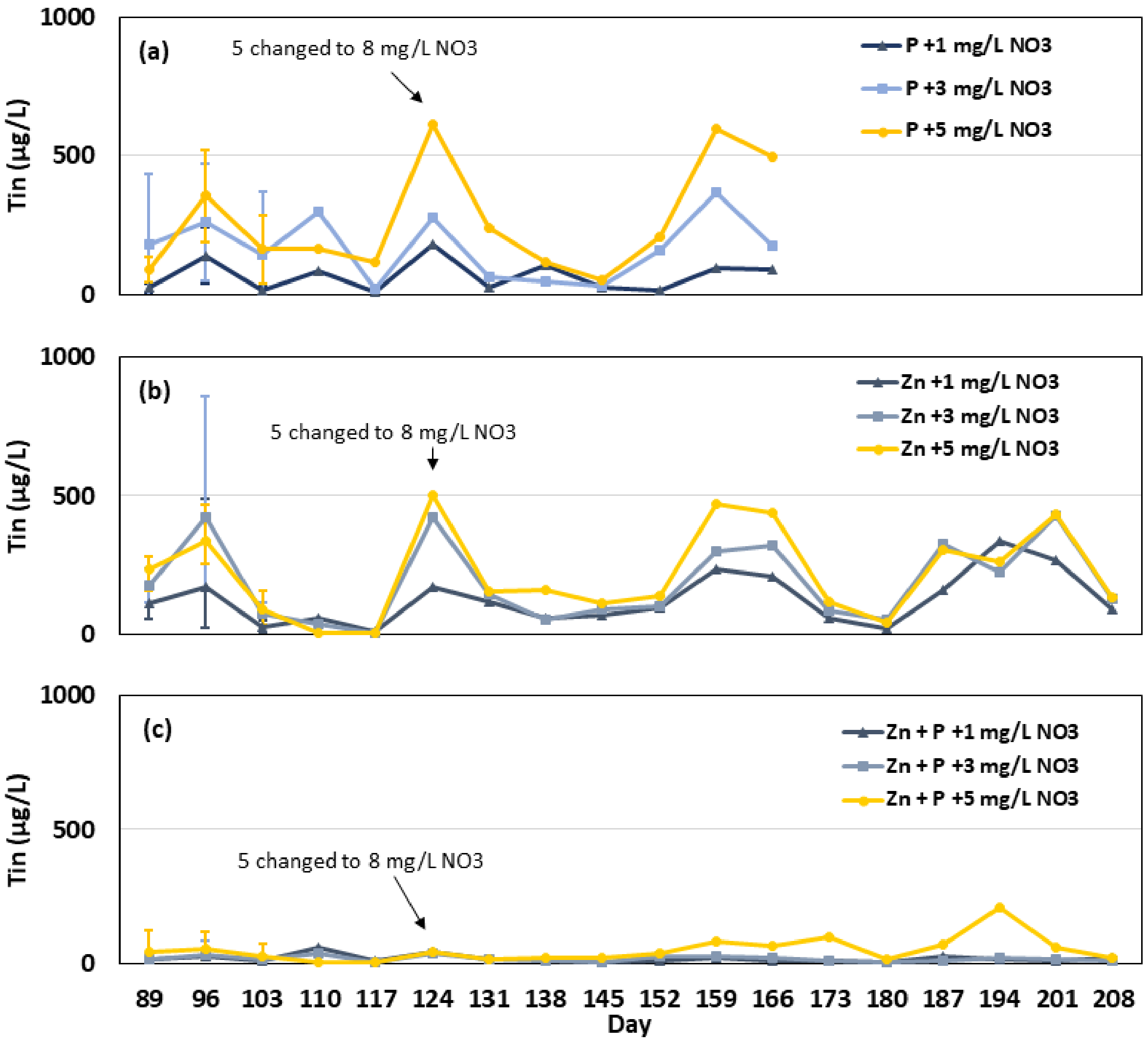

SI Figure 5a-c. Tin release during Phases 2-4 from coupons treated with a) orthophosphate, b) zinc, and c) zinc orthophosphate with various levels of nitrate. Coupons that were receiving 5 mg/L NO_3-N began receiving 8 mg/L NO_3-N on day 124 (Phase 3). Error bars represent measurement ranges. After day 103, composite samples were collected (n=5).

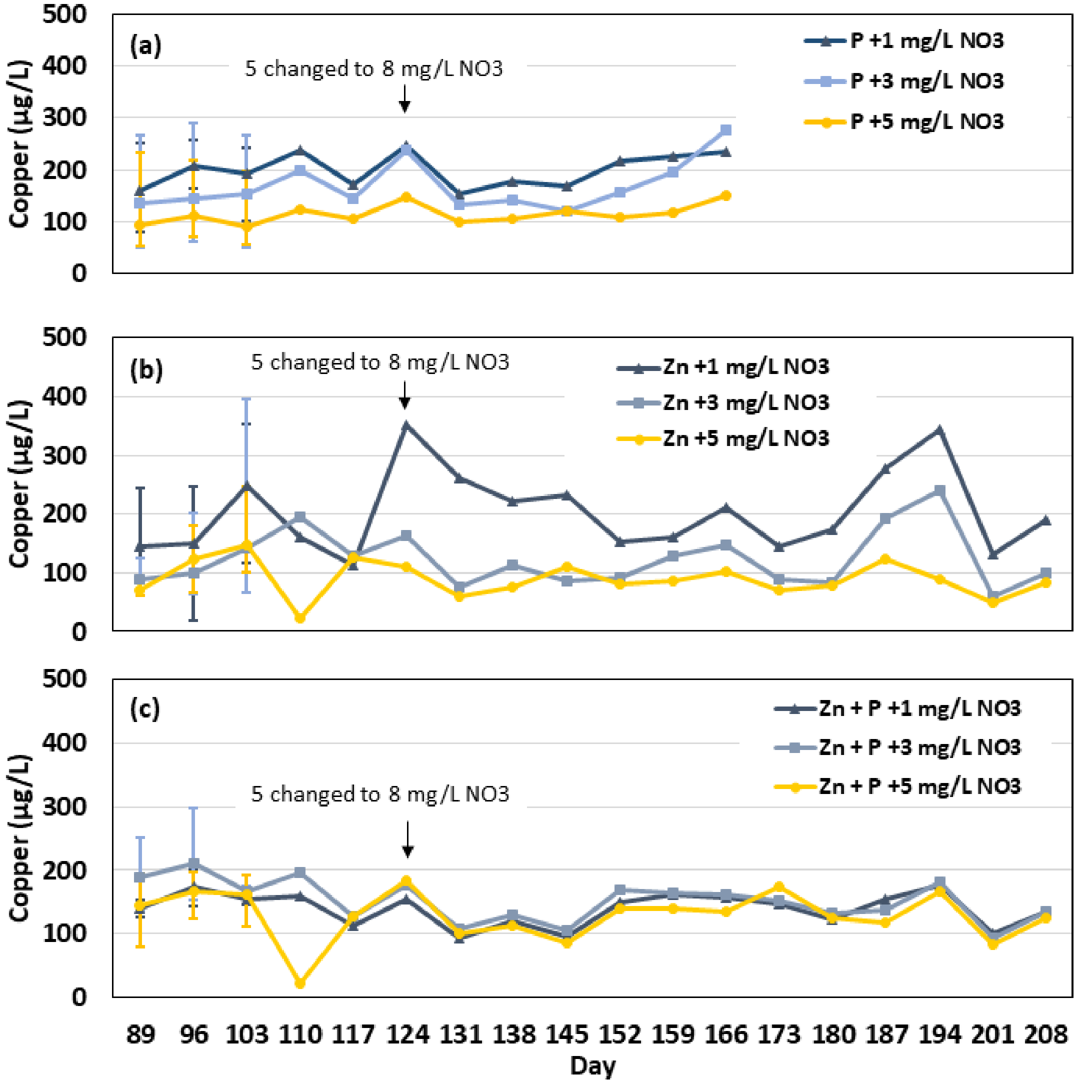

SI Figure 6a-c. Copper release during Phases 2-4 from coupons treated with a) orthophosphate, b) zinc, and c) zinc orthophosphate and various levels of nitrate. Coupons that were receiving 5 mg/L NO_3-N began receiving 8 mg/L NO_3-N on day 124 (Phase 3). Error bars represent measurement ranges. After day 103, composite samples were collected (n=5).

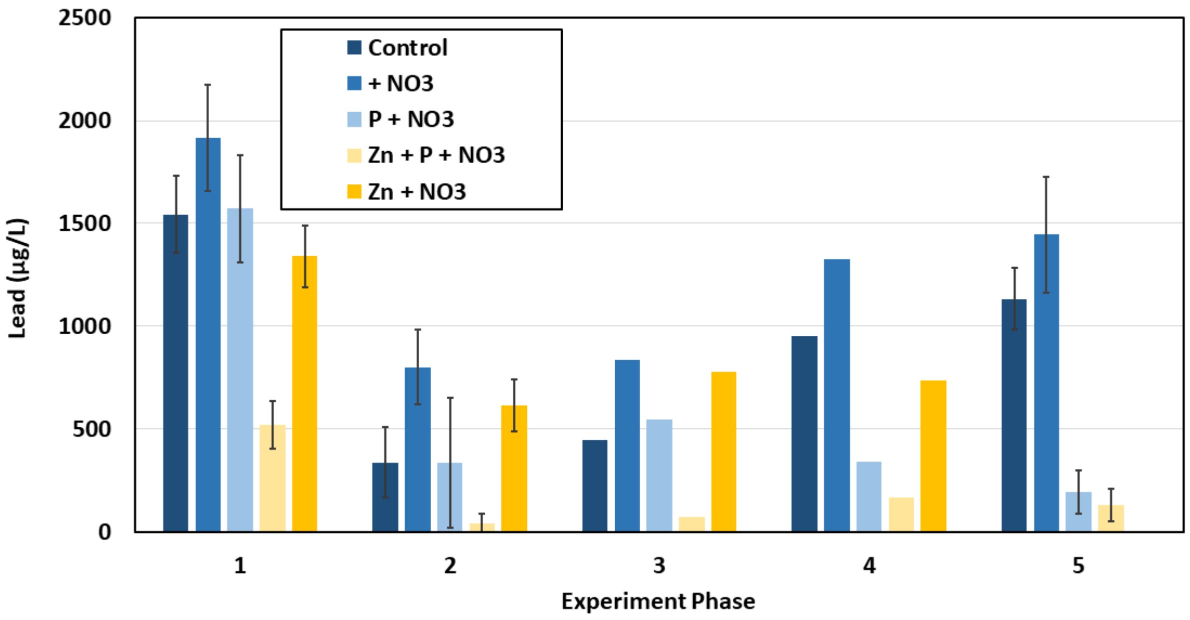

SI Figure 7. Average lead release from the 5 phases of the coupon study. In Phase 2, +NO3 refers to the condition subgroups receiving +5 mg/L NO_3-N (Groups D, I, L, and O). From Phase 3 onwards, the +5 mg/L NO_3-N dose was increased to +8 mg/L NO_3-N. In Phase 4, the orthophosphate condition became zinc orthophosphate (0.66 mg/L Zn) and the zinc condition was removed in Phase 5. Error bars represent 95% confidence intervals and composite samples were collected during Phases 3 and 4.

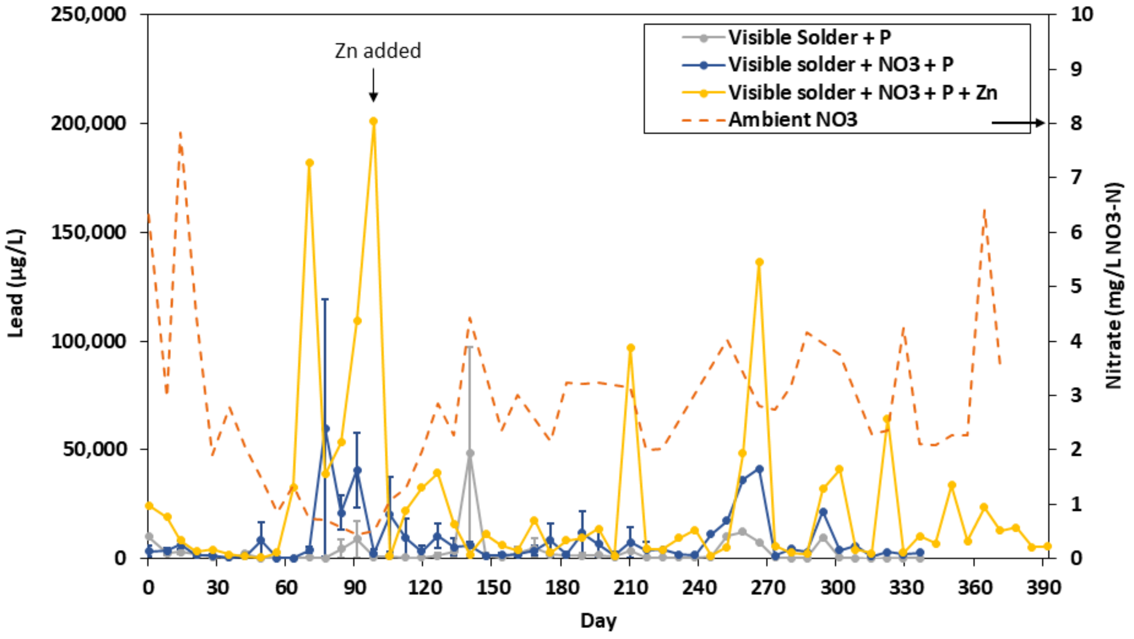

SI Figure 8. Lead release from the harvested pipes with visible solder (Pipes E, F, and G) and ambient nitrate levels versus time. Nitrate was added to select pipes (Pipes F and G) on day 56 following a conditioning phase. Zinc orthophosphate was added to one pipe (Pipe G) on day 98. All pipes received 1 mg/L orthophosphate as P. Error bars represent measurement ranges. Adapted from "Seasonal fluctuations in nitrate levels can trigger lead solder corrosion problems in drinking water," by Lopez, K. et al., 2023, *Environ. Sci. Technol. Lett.* **10**, p. 21-26. Copyright 2023 American Chemical Society.

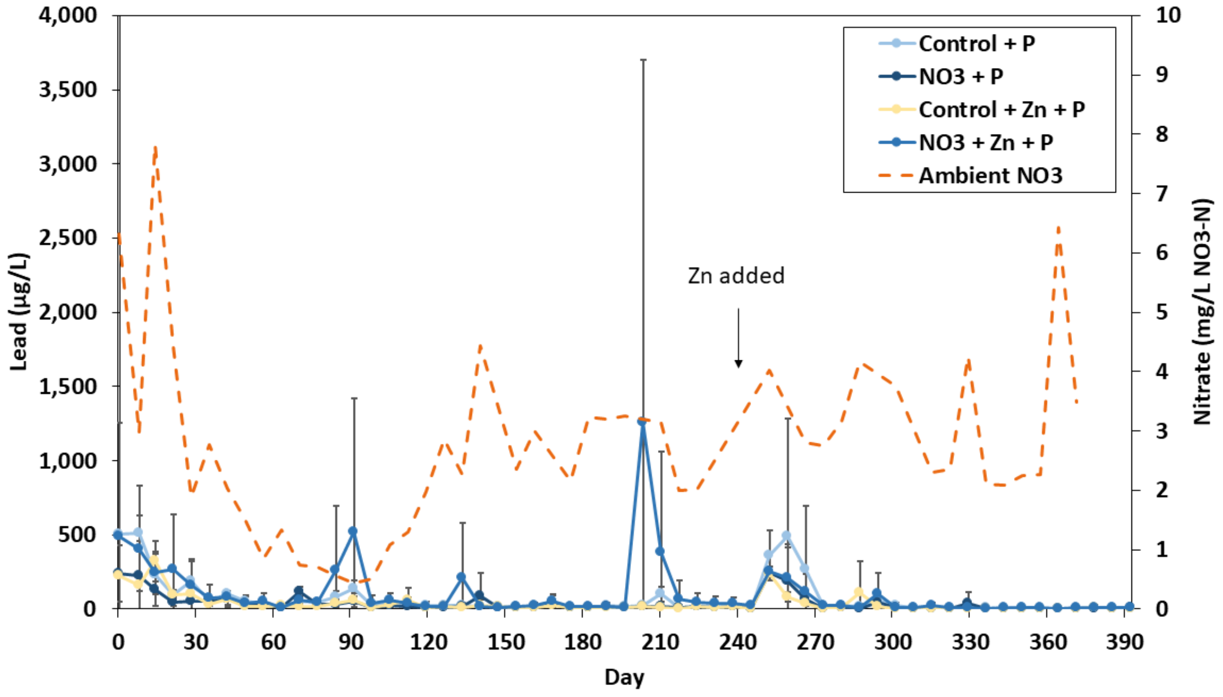

SI Figure 9. Lead release from the harvested pipes without visible solder and ambient nitrate levels versus time. Nitrate was added to select pipes on day 56 following a conditioning phase, and zinc orthophosphate was added to select pipes on day 252. All pipes received 1 mg/L orthophosphate as P. Error bars represent 95% confidence intervals. Adapted from "Seasonal fluctuations in nitrate levels can trigger lead solder corrosion problems in drinking water," by Lopez, K. et al., 2023, *Environ. Sci. Technol. Lett.* **10**, p. 21-26. Copyright 2023 American Chemical Society.

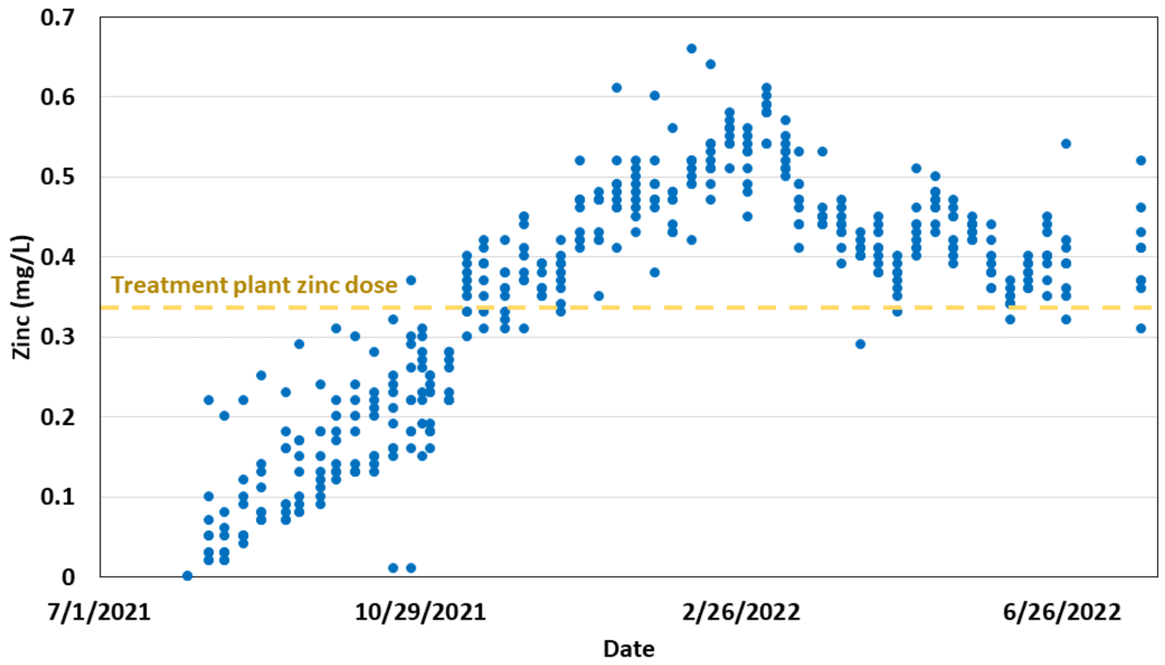

SI Figure 10. Zinc levels in the distribution system increase over time. Each time point shows multiple sampling locations, including those near and far from the water treatment plant. 0.33 mg/L zinc was added to the system in August of 2021 (indicated by the yellow line) and data was supplied by water utility.

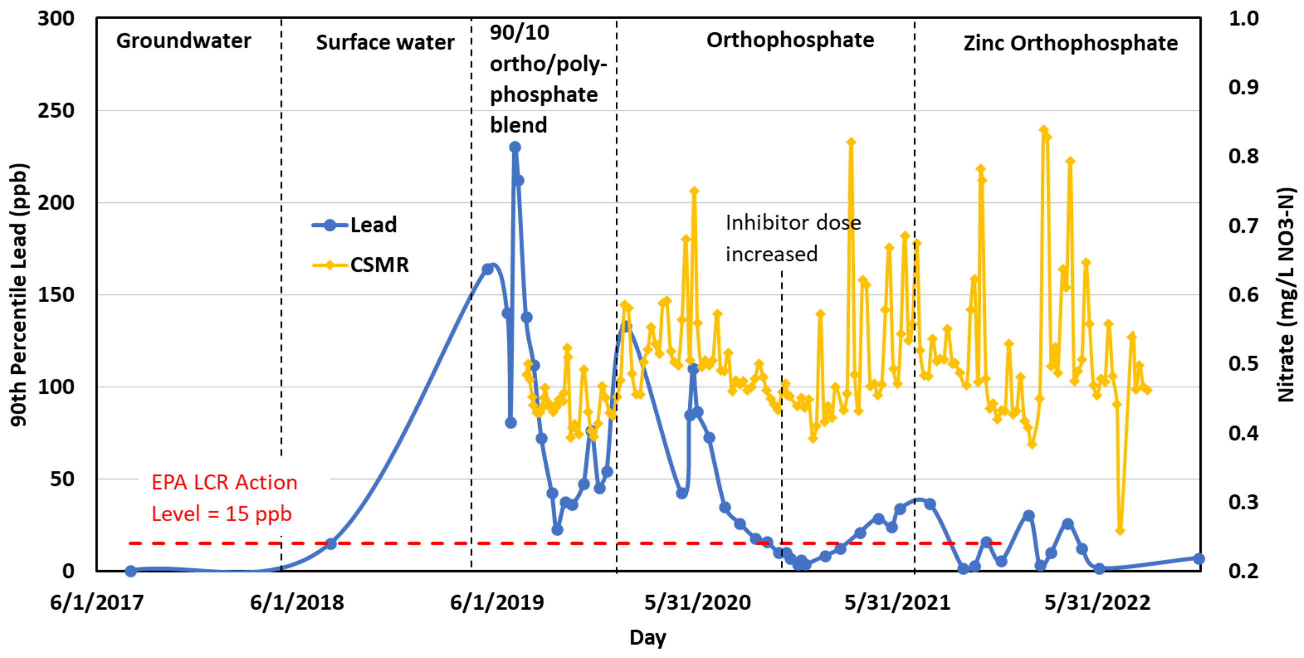

SI Figure 11. 90th percentile lead release and fluctuations in CSMR over time in the affected community. Changes in corrosion control are noted. Adapted from "Seasonal fluctuations in nitrate levels can trigger lead solder corrosion problems in drinking water," by Lopez, K. et al., 2023, *Environ. Sci. Technol. Lett.* **10**, p. 21-26. Copyright 2023 American Chemical Society.

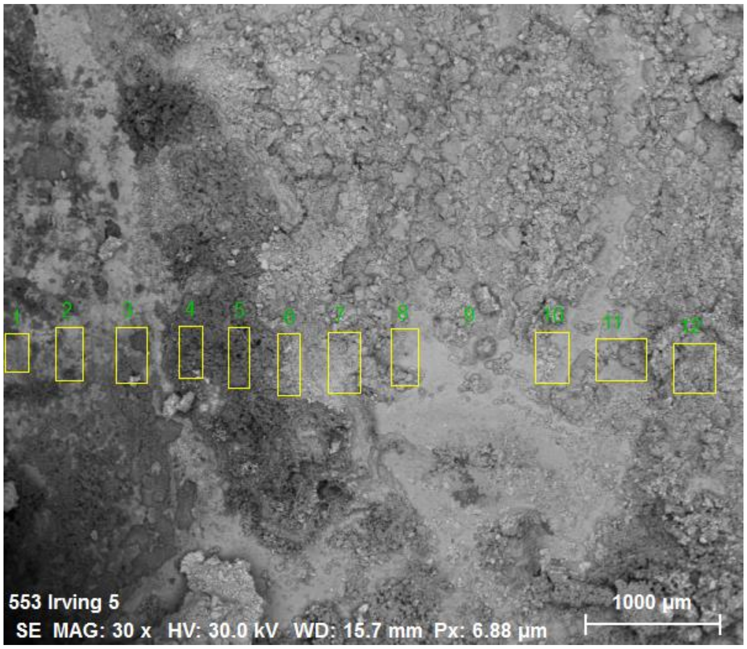

SI Figure 12. Region of harvested pipe treated with zinc orthophosphate prior to extraction that was analyzed with SEM.
